# Production, Purification, and Study of the Amino Acid Composition of Microalgae Proteins

**DOI:** 10.3390/molecules26092767

**Published:** 2021-05-08

**Authors:** Anna Andreeva, Ekaterina Budenkova, Olga Babich, Stanislav Sukhikh, Elena Ulrikh, Svetlana Ivanova, Alexander Prosekov, Vyacheslav Dolganyuk

**Affiliations:** 1 Institute of Living Systems, Immanuel Kant Baltic Federal University, 236016 Kaliningrad, Russia; AnnaAndreeva@kantiana.ru (A.A.); abudenkova@kantiana.ru (E.B.); olich.43@mail.ru (O.B.); stas-asp@mail.ru (S.S.); dolganuk_vf@mail.ru (V.D.); 2 Department of Bionanotechnology, Kemerovo State University, 650043 Kemerovo, Russia; 3 Kuzbass State Agricultural Academy, 650056 Kemerovo, Russia; elen.ulrich@mail.ru; 4 Natural Nutraceutical Biotesting Laboratory, Kemerovo State University, 650043 Kemerovo, Russia; 5 Department of General Mathematics and Informatics, Kemerovo State University, 650043 Kemerovo, Russia; 6 Laboratory of Biocatalysis, Kemerovo State University, 650043 Kemerovo, Russia; a.prosekov@inbox.ru

**Keywords:** microalgae, protein concentrate, purification, ultrafiltration, amino acid composition

## Abstract

Microalgae are known to be rich in protein. In this study, we aim to investigate methods of producing and purifying proteins of 98 microalgae including *Chlorella vulgaris, Arthrospira platensis*, *Nostoc* sp., *Dunaliella salina*, and *Pleurochrysis carterae* (Baltic Sea). Therefore, we studied their amino acid composition and developed a two-stage protein concentrate purification method from the microalgae biomass. After an additional stage of purification, the mass fraction of protein substances with a molecular weight greater than 50 kDa in the protein concentrate isolated from the biomass of the microalga *Dunaliella salina* increased by 2.58 times as compared with the mass fraction before filtration. In the protein concentrate isolated from the biomass of the microalga *Pleurochrysis cartera*, the relative content of the fraction with a molecular weight greater than 50.0 kDa reached 82.4%, which was 2.43 times higher than the relative content of the same fractions in the protein concentrate isolated from this culture before the two-stage purification. The possibilities of large-scale industrial production of microalgae biomass and an expanded range of uses determine the need to search for highly productive protein strains of microalgae and to optimize the conditions for isolating amino acids from them.

## 1. Introduction

The total protein content in the biomass of microalgae depends on the type of microalgae and can reach 70% of the dry weight [1,2]. The cell walls of microalgae are often destroyed to ensure access to proteins, amino acids, and other components. It has been reported that some microalgae contain soluble proteins in their cytoplasm [3,4]. In addition, microalgae with chloroplasts contain soluble protein, central pyrenoid, and phytobiliproteins, although some microalgae, such as *Arthrospira platensis*, instead, have thylakoid sacs surrounding the peripheral cytoplasm associated with phycobilisomes [5,6].

The number of studies on methods of processing microalgae and using them as a source of protein has rapidly increased in recent years [7]. Vernèsa et al. [8] proposed a new method of protein extraction from *Arthrospira platensis* based on the combined effect of three parameters: pressure, temperature, and ultrasound. When using the developed method, the authors managed to increase the protein yield by 229% as compared with the conventional method of ultrasonic exposure. It was assumed that the combined effect of pressure, temperature, and ultrasound was better at destroying cells and intensifying the process of mass transfer as compared with using only ultrasound. Acoustic cavitation affected *Arthrospira platensis* filaments through various mechanisms such as fragmentation, sonoporation, and destruction. These phenomena contributed to the more efficient extraction of proteins from *Arthrospira platensis*.

Many methods for concentrating and isolating proteins from microalgae are difficult to scale up. The three-phase separation method has attracted the interest of many researchers due to its fast, simple, and scalable use for concentrating, isolating, and deactivating proteins from crude samples. Waghmare et al. [9] studied the effect of various parameters on the three-phase separation method to optimize the process of protein isolation from *Chlorella pyrenoidosa*. Protein extraction from microalgae *Chlorella vulgaris* by the method of three-phase separation with sonication was presented in their work. As a result of using the additional ultrasonic treatment, the authors managed to obtain an increased protein yield in a shorter period of time. It is assumed that ultrasonic three-phase separation is a more efficient method for extracting biomolecules from microalgae [9].

Chia et al. [10] proposed a method of microwave three-phase separation of proteins of microalga *Chlorella vulgaris*, which was an improved version of the traditional three-phase separation method. The authors optimized the conditions for applying the method as follows: concentration of ammonium sulfate (30% *w/w*), suspension to solvent ratio (1:1), microwave radiation time (120 s), duty cycle (80%), microwave radiation power (100 W), and the concentration of biomass of microalgae (0.5% *w/w*). The developed method made it possible to increase protein yield by 2.54 times as compared with the usual three-phase separation. Microwave radiation promoted deeper destruction of microalgae cells [11].

Grimi et al. [12] analyzed the efficiency of water extraction of proteins from five species of microalgae (*Haematococcus pluvialis, Nannochloropsis oculata, Chlorella vulgaris, Porphyridium cruentum*, and *Arthrospira platensis*) using various methods of cell destruction. They found that the highest yield of protein under the conditions of water extraction occurred when cells were destroyed under high pressure, followed by chemical treatment, ultrasonic treatment, and mechanical treatment [13].

A method for the extraction of proteins and carbohydrates from *Spirulina platensis* biomass using ultrasonic treatment and mechanical stirring under alkaline conditions was presented in [14]. Under optimized extraction conditions with sonication for 33–40 min and stirring for 40–55 min, the yield of proteins was 75.76%, and the yield of carbohydrates was 41.52%.

In [15], the authors presented a gentle process of bioprocessing of the microalga *Nannochloropsis gaditana* to obtain a water-soluble protein fraction free from chlorophyll. To destroy cells, homogenization under pressure or enzymatic hydrolysis was used, followed by ultrafiltration/diafiltration.

The authors of [16] evaluated the effect of various types of solvents (methanol, ethanol, 1-propanol, and water) on the release of proteins from the cell wall of microalgae. They found that water was the most effective extractant of microalgae proteins as compared with other solvents.

It is known that algae such as *Chlorella vulgaris, Arthrospira platensis, Nostoc* sp., *Dunaliella salina*, and *Pleurochrysis carterae* quickly build up biomass and accumulate useful components; are unpretentious to cultivation conditions and the composition of nutrient media; and are suitable for use in the food, feed, cosmetic, and pharmaceutical industries. Extraction of valuable components, including protein, requires a suitable technology that provide qualitative and quantitative yield of the target product. Traditional separation methods (membrane separation, column chromatography, precipitation, and crystallization) of microalgal proteins often consist of several sequential operations, require multiple repetitions, and use a large amount of toxic organic solvents. This is reflected in increased time and economic costs, including product loss throughout the entire process.

In this study, we investigate methods for obtaining and purifying proteins of microalgae *Chlorella vulgaris, Arthrospira platensis, Nostoc* sp., *Dunaliella salina*, and *Pleurochrysis carterae* and we study their amino acid composition.

## 2. Results

**Figure 1:**
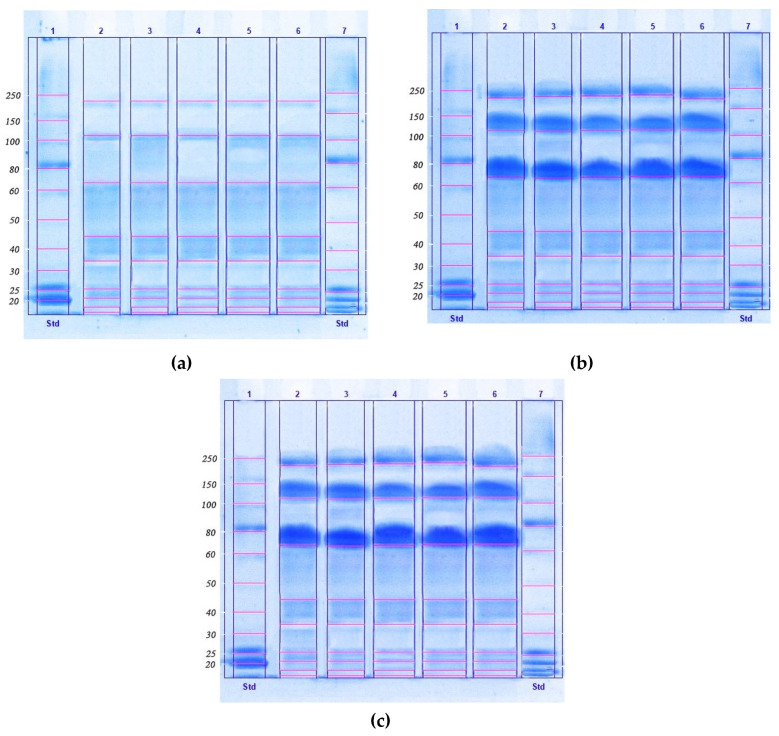
The result of obtaining protein isolates from microalgal biomass (**a**), microalgal biomass after the ultrafiltration process (**b**), and microalgae biomass after HPLC (**c**), by electrophoresis in polyacrylamide gel (12%) in the presence of sodium dodecyl sulfate (SDS-PAGE).

The method we developed involved a two-stage purification scheme, as a fast, simple, and scalable method for concentrating, isolating, and disinfecting proteins from lyophilized biomass of microalgae, providing a high yield and clean extraction. In the first stage of this study, a method was developed for purifying the protein concentrate isolated from the biomass of microalgae (*Chlorella vulgaris*, *Arthrospira platensis*, *Nostoc* sp., *Dunaliella salina*, and *Pleurochrysis carterae*), which was used to isolate the protein complex from the biomass. Figure 1 demonstrates the SDS-PAGE of protein isolates from the microalgal biomass.

**Figure 2:**
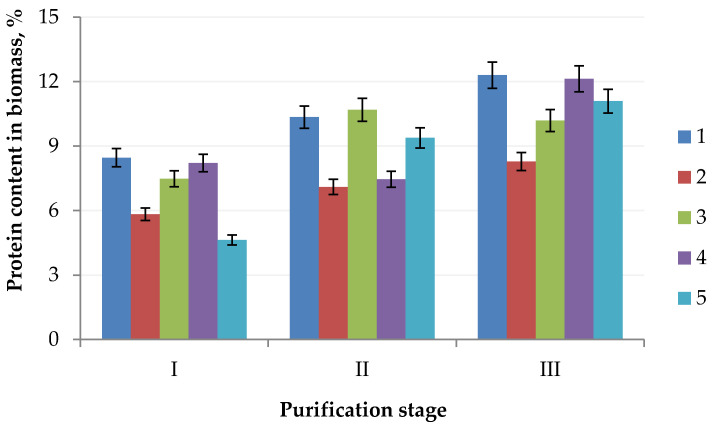
Change of the protein content in the algae biomass: (1) *Chlorella vulgaris*; (2) *Arthrospira platensis*; (3) *Nostoc* sp.; (4) *Dunaliella salina*; (5) *Pleurochrysis carterae*) in the purification process (stage I, extraction; stage II, ultrafiltration; stage III, HPLC).

To produce a protein concentrate, which can be used in the future as a food or feed ingredient, from the biomass of microalgal cell cultures, it is necessary to consider that the protein concentrate must contain proteins with a molecular weight greater than 50.0 kDa. The presence of protein substances with a molecular weight less than 50.0 kDa in the product is the reason for a decrease in organoleptic characteristics, i.e., the appearance of a bitter taste and unpleasant odor, which is unacceptable due to the use of a protein concentrate as a food and/or feed ingredient [17]. We found that *Chlorella vulgaris* contains 12.30% protein, *Arthrospira platensis* contains 8.28% protein, *Nostoc* sp. contains 10.19% protein, *Dunaliella salina* contains 12.13% protein, and *Pleurochrysis carterae* contains 11.09% protein (Figure 2).

In this regard, we studied the molecular weight distribution of protein substances and polypeptides of the protein complex isolated from the biomass of microalgal cell cultures, and the results are shown in Table 1.

A detailed analysis of the results given in Table 1 allowed us to establish that the isolated protein complex must be purified from low molecular weight compounds. The possibility of using two purification schemes for the protein concentrate was assessed:

– a one-stage method of ultrafiltration;
– a two-stage method of ultrafiltration and high-performance liquid chromatography (HPLC).

For most of the microalgae samples, there were no statistically significant differences in the size of protein fractions (*p* > 0.05), except for *Nostoc* sp. and *Dunaliella satina*. For samples of protein fractions with a molecular weight greater than 50.0 kDa, statistically significant differences (*p* < 0.05) were revealed only for the microalgae *Nostoc* sp. After the first phase, the proportion of protein fractions with a molecular weight greater than 50.0 kDa for all microalgae samples did not exceed 34%.

The protein concentrate was ultrafiltered to improve quality characteristics. Preliminary studies have shown that the ultrafiltration of protein substances isolated from the biomass of microalgae cell cultures is most effective when using membranes with a pore diameter of 50.0 kDa at an active acidity of 7.0 and a process pressure of 0.2 MPa. In this ultrafiltration mode, the integral selectivity of the membrane for protein was 88–97%, and the degree of concentration reached 5.63–5.81 for all microalgae samples.

To assess the efficiency of the ultrafiltration process of the protein complex isolated from the biomass of cell cultures, the protein concentrate was fractionated. The results are shown in Table 2.

Statistically significant differences in the size distribution of protein fractions were observed for almost all values of the samples obtained for *Dunaliella satina* and *Pleurochrysis carterae*. For protein fractions with a molecular weight greater than 50 kDa, statistically significant differences (*p* < 0.05) were identified for all microalgae samples with a clear predominance of the above species. The analysis of the results of studying the fractional composition of the protein complex obtained from the microalgae biomass after the ultrafiltration process indicates that this method concentrates the protein and increases the content of high-molecular protein substances with a molecular weight greater than 50.0 kDa. The proportion of protein fractions with a molecular weight greater than 50.0 kDa was from 52 to 60% for the studied microalgae samples. The most significant increase, i.e., 1.85 times, was observed in the *Dunaliella salina* samples.

Then, ultra-concentrated samples of the protein concentrate were used at the next stage for the removal of low molecular weight protein fractions by high-performance liquid chromatography. The results are shown in Table 3.

After the additional stage of purification of the protein concentrate by HPLC (Table 3), statistically significant differences (*p* > 0.05) between algal samples for protein complexes with molecular weights from 13.0 to 15.0 kDa, from 20.0 to 21.0 kDa, and from 33.0 to 35.0 kDa were not found. Statistically significant differences in protein complexes of molecular weight greater than 70.0 kDa were found for the samples of *Arthrospira platensis* (*p* = 0.042), *Dunaliella salina* (*p* = 0.016–0.040), and *Pleurochrysis carterae* (*p* = 0.007–0.032). However, for the protein concentrate obtained from *Nostoc* sp., reduced content of the fraction with a molecular weight of 27.0–25.0 kDa was noted as compared with other species. In addition, an increased content of the 70.0–67.0 kDa fraction was observed after HPLC for the same species. The share of the protein fraction with a molecular weight greater than 50.0 kDa increased. We found that the share of the fraction with a molecular weight from 67.0 to 227.0 kDa in the protein concentrate obtained from the *Chlorella vulgaris*, *Arthrospira platensis, Nostoc* sp., *Dunaliella salina*, and *Pleurochrysis carterae* microculture after the stage of concentration by ultrafiltration and HPLC was 77.5%, 78.9%, 80.8%, 80.8%, and 82.4%, respectively.

The mass fraction of protein fractions with a molecular weight greater than 50.0 kDa after the third stage of purification increased by 1.4–1.5 times, and approximately 2.4–2.5 times as compared with the initial value. In all the microalgae, the total amount of protein with a molecular weight greater than 50.0 kDa obtained at different stages of purification was approximately the same. The selection criterion for the preferred species of microalgae as a source of protein can be the qualitative characteristics of the obtained concentrates. Since the biological value of the protein component of food and raw materials depends on the qualitative and quantitative amino acid composition, we further studied the amino acid profile of proteins in microalgae. Amino acids were analyzed in the microalgae protein complex after ultrafiltration and HPLC. In this case, the sum of all amino acids obtained by SDS-PAGE is taken as 100 g of protein. The results are shown in Table 4.

Samples of protein concentrates from *Chlorella vulgaris* and *Dunaliella salina* algae are almost identical. Statistically significant differences in amino acid profiles were found in samples of *Arthrospira platensis* (aspartic acid, glutamic acid, glycine, lysine, threonine, tryptophan, and valine), *Nostoc* sp. (glycine, tryptophan, and valine), and *Pleurochrysis carterae* (glutamic acid, lysine, tryptophan, and valine).

## 3. Discussion

Microalgae *Chlorella vulgaris, Arthrospira platensis, Nostoc* sp., *Dunaliella salina*, and *Pleurochrysis carterae* were selected for research on producing and purifying proteins and studying their amino acid composition since they are the source of simple proteins (about 50%). Studies [6,18,19] have shown that these microalgae are rich in minerals, vitamins, amino acids, antioxidants, and oncoprotectors, as well as have a sugar-lowering effect, which explains their widespread use in various fields of human activity: medicine, cosmetics, sports, animal husbandry, beekeeping, fish and poultry farming, and veterinary medicine.

The method used with additional purification steps increased the content of protein fractions with a molecular weight greater than 50.0 kDa. Protein concentrates obtained from the microalgae *Chlorella vulgaris* (12.30%) and *Dunaliella salina* (12.13%) were distinguished by the maximum protein content after three-stage purification. The minimum protein content (8.28%) was observed in the protein concentrate obtained from *Arthrospira platensis*. This fact is likely associated with the different structures of cells in algae, which affects the process of protein extraction from them.

Kai Ru et al. [12] presented results of the fractionation of protein concentrate of microalgae biomass that were comparable to ours. In particular, [12] showed that the share of the fraction with a molecular weight from 67.0 to 227.0 kDa in the protein concentrate obtained from the *Chlorella vulgaris* microculture was 76.9%, in the protein concentrates obtained from the biomass of the *Arthrospira platensis* and *Nostoc* sp. microalgae, these proteins were 79.1% and 80.6%, respectively. The share of high-molecular-weight proteins in protein concentrates obtained from the biomass of microalgae *Dunaliella salina* and *Pleurochrysis carterae* also exceeded the values we obtained, i.e., 58.7 and 82.4%, respectively. The similarity of the research results [5,12,13] with our results is explained by the fact that a sufficiently high content of proteins in microalgae allows one to use various methods of isolating and concentrating proteins and determining their amino acid composition.

To meet the daily human requirement for amino acids, it is sufficient to consume 5–20 g of a microalgae product after ultrafiltration and HPLC [6]. All microalgae proteins have a caloric content of 75–86 kcal/100 g, which determines the significant nutritional value of these proteins [20]. For concentrates obtained from *Chlorella vulgaris, Arthrospira platensis, Nostoc* sp., *Dunaliella salina*, and *Pleurochrysis carterae*, the total contents of essential amino acids (valine, isoleucine, leucine, lysine, methionine, threonine, tryptophan, and phenylalanine) were 33.02, 42.00, 44.72, 42.45, and 45.85 g/100 g of concentrate, respectively. Soybeans, one of the richest protein products, have a similar protein content, i.e., 12.59 g/100 g. In terms of all essential amino acids, the samples of protein concentrates of the studied algae species surpass other protein sources of both animal and plant origin in quantitative and qualitative content.

## 4. Materials and Methods

### 4.1. Materials and Chemicals

The microalgae samples were collected, as described in [21]. For this, we used pure phytoplankton collected by nets with appropriate cells. The upper part of the net is water permeable. The microalgae caught in the net were concentrated at the bottom of the container. After preliminary washing on the filter, microalgae were placed in a bottle with water (about 50 mL), and concentrated samples were delivered to the laboratory. Three mL of microalgae concentrate was placed in a petri dish. To identify microalgae in the sample, we examined them under a microscope every 2 h and established the presence of threads and colonies of microalgae of a certain species. An MIKMED-6 microscope with an imaging system (LOMO JSC, St. Petersburg, Russia) was used. The micrographs were processed using the FOTO Microanalysis software (LOMO JSC, St. Petersburg, Russia) and Levenguk LabZZ (Levenhuk, Tampa, FL, USA). A light intensity of no more than 500 lux was used [22].

Partial sequences of the 18S and/or 16S rRNA gene (Appendix A, Appendix B, Appendix C, Appendix D and Appendix E) were determined to identify isolated microalgae from the enriched culture, followed by a comparison with known sequences from the GenBank database.

The objects of research were microalgae *Chlorella vulgaris, Arthrospira platensis, Nostoc* sp., *Dunaliella salina*, and *Pleurochrysis carterae*, from which a protein concentrate was produced. Microalgae were collected in the Baltic Sea in 2020. We used reagents of analytical grade and higher.

### 4.2. Microalgae Cultivation

*Chlorella vulgaris* strains were cultured on Tamiya medium; for the cultivation and biomass production of the *Arthrospira platensis* microalgae, the Zarrouk’s nutrient medium was used; for the production of *Dunaliella salina* biomass, the Omarov’s medium was used. Nutrient medium BG-11 was used for *Nostoc* sp. cultivation, F/2 nutrient medium was used for the production of *Pleurochrysis carterae* biomass, and a special nutrient medium was used for *Chlorella vulgaris*. The culture media were sterilized by autoclaving, the microelements of the Zarrouk’s medium were sterilized by filtration through a filter with a pore diameter of 0.22 μm and added after autoclaving into culture media cooled to room temperature.

### 4.3. Production of Protein Concentrate

The Bradford method was used to obtain highly concentrated protein [23]. For protein extraction, 10 mL of 0.5 M sodium hydroxide solution was added to 10 mg of dry microalgae biomass and kept in a water bath for 10 min at 80 °C. The resulting extract was centrifuged for 20 min at a rotor speed of 3900 rpm. The supernatant was transferred to a clean chemical ware.

The fractional composition of proteins in the obtained protein concentrate was determined by the method of molecular weight distribution.

This method is based on the shift of the absorption maximum of the optical density of the acid blue 90 (Coomassie Brilliant Blue R-250, Sigma-Aldrich Rus, Moscow, Russia) dye from 470 nm to 595 nm, observed due to the binding of the protein to the dye. The dye most actively binds to the arginine and lysine residues of the protein, which can lead to errors in the quantitative determination of various types of proteins. The protein used as a reference material was the same as the protein under test. The appropriate standard protein sample was dissolved in the buffer solution. Parts of the resulting solution were diluted in the same buffer solution to obtain at least five standard solutions with protein concentrations uniformly distributed in the range between 0.1 and 1 mg/mL (used to construct a calibration graph).

Bradford reagent (5 mL, Sigma-Aldrich Rus, Moscow, Russia) was added to 0.1 mL of each standard, test, and control solution. They were mixed thoroughly by inverting. Foam formation resulting in poor reproducibility was avoided. They were kept at room temperature for 10 min, and then the optical densities of the standard solutions and the test solution were measured on a spectrophotometer at a wavelength of 595 nm, using the control solution as a reference solution containing solvent and Bradford reagent. The spectrophotometric cuvettes must not be quartz ones. The color remained stable for 1 h. A calibration graph of the dependence of the optical densities of standard solutions on protein concentrations was constructed; linear regression was used. The protein concentration in the test solution was determined based on the calibration curve and the optical density of the test solution.

Lyophilized biomass of microalgae was used for protein extraction. Lyophilization was carried out using an AK apparatus (Proflab, St. Petersburg, Russia) for 24 h, at a pressure of 0.5 bar and a condenser temperature of minus 50 °C, in one stage without shelf heating. Then, 10 mL of 0.5 M sodium hydroxide solution was added to 10 mg of lyophilized microalgae biomass and kept in a water bath for 10 min at 80 °C. The obtained extract was centrifuged for 20 min at a rotor speed of 3900 rpm, a temperature of 25 °C, and a maximum centrifugal acceleration of 2000 g. The supernatant was transferred to a clean chemical container [24].

### 4.4. Purification of Protein Concentrate

Ultrafiltration of the protein concentrate was carried out through membranes with pore diameters of 50.0 and 100.0 kDa at different values of active acidity and pressure. The active acidity varied in the range from 5.0 to 9.0 with a step of 1.0. The efficiency of the one-stage ultrafiltration process was assessed by the integral membrane selectivity and the degree of concentration [25].

HPLC was performed on an LC-20 chromatograph (Shimadzu, Kyoto, Japan), eluting protein concentrate samples in a sodium chloride concentration gradient. Detection was carried out using a diode array detector in the detection range of 180–900 nm, the flow rate of the eluent in all cases was one mL/min, elution was carried out in a gradient mode, the time and gradient were selected individually for each case of separation, the mixture of treated water (MQ purification level) and acetonitrile with the addition of 0.1% trifluoroacetic acid was used as solvents, separation was carried out on a reversed-phase Phenomenex column (Torrance, CA, USA) 250 mm × 2.5 mm, particle size 25 μm, sorbent was silica gel modified C-18, with phenyl end-capping [26].

After additional purification of the protein concentrate by HPLC, the fractional composition of proteins was determined by the method of molecular weight distribution.

### 4.5. Determination of the Protein Concentrate Amino Acid Composition

**Scheme 1:**
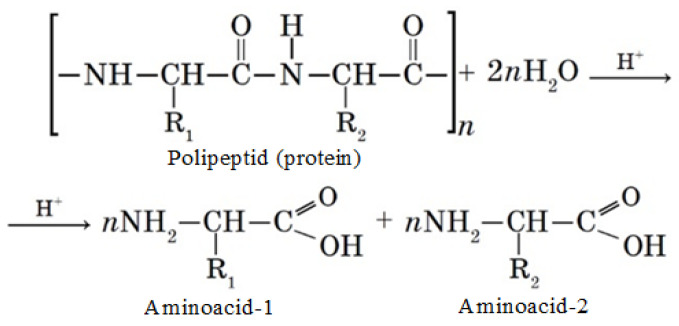
Protein hydrolysis process.

To study the amino acid composition of proteins, a method was used based on the decomposition of the objects of study under the action of acids (concentrated hydrochloric and sulfuric acids) and inorganic alkali into free forms of amino acids, their further separation, and quantitative determination of the latter on a capillary electrophoresis system Capel-105/105M (Lumex, St. Petersburg, Russia) using the M-04-38-2009 technique, which is based on the extraction of acids from the test sample with distilled water, separation, and quantitative determination of components by capillary electrophoresis with indirect detection at a wavelength of 190 nm [27]. An L-amino acids kit LAA-21 by Lumex (Lumex, St. Petersburg, Russia) was used as standard samples. The protein hydrolysis process in the presence of acid (H^+^) is shown in Scheme 1.

### 4.6. SDS-PAGE Analysis

SDS-PAGE was performed according to the method described by Schägger and Von Jagow [28] using 4% stacking gel (*w/v*) and 12% polyacrylamide gel (*w/v*). First, 10 milligrams of protein isolate was dissolved in 1 mL of denaturing buffer for samples, (0.5 M Tris-HCl pH 6.8, glycerol, 10% SDS, 0.5% bromophenol blue, β-mercaptoethanol) and heated at 95 °C. Then, 10 μL of the sample was loaded into the sample wells. Protein separation was performed at 80 V for 30 min, then at 110 V for 90 min for a separation gel using a Mini Protean II device (Bio-Rad Laboratories, Hercules, CA, USA). The gel was stained with brilliant blue for 40 min (Bio-Rad Coomassie R250, Bio-Rad Laboratories, Hercules, CA, USA). Gel bleaching was performed three times using water/methanol/acetic acid (7/2/1 *v/v/v*) for 15 min each shaking cycle using an orbital shaker (Fristek S10, Taichung, Taiwan). The molecular weight of proteins was evaluated using a protein molecular weight marker (250 to 10 kDa, Bio-Rad Laboratories, Hercules, CA, USA) loaded at a dose of 5 μL into the sample well. Gels were scanned with an E-Box VX5 (Vilber Lourmat, Paris, France), and captured image analysis was performed using Vision Capt software (V16.08a, Vilber Lourmat, Paris, France).

### 4.7. Statistical Analysis

Each experiment was repeated three times, and the data are expressed as means ± standard deviation. Post hoc analysis (Duncan test) was undertaken to identify significantly different samples from each other. The equality of the variances of the extracted samples was checked using the Levene test. The data were subjected to analysis of variance (ANOVA) using Statistica 10.0 (StatSoft Inc., Tulsa, OK, USA, 2007). Differences between means were considered significant when the confidence interval was below 5% (*p* < 0.05).

## 5. Conclusions

For the first time, a screening of the amino acid profile of proteins contained in microalgae (*Chlorella vulgaris, Arthrospira platensis, Nostoc* sp., *Dunaliella salina*, and *Pleurochrysis carterae*) was carried out. In the framework of this research, a method for purifying protein concentrate obtained from the biomass of cell cultures of microscopic algae was developed, which includes the process of ultrafiltration (active acidity 7.0, process pressure 0.2 MPa, and a membrane with a pore diameter of 50.0 kDa) followed by HPLC in a sodium chloride concentration gradient. This method is not destructive for other biologically active substances (lipids, carbohydrates, macro- and microelements, vitamins, bioflavonoids, tannins, and other BAS) contained in microalgae [24,25]. Existing technologies can isolate 60% of the protein from algae biomass in industrial conditions, up to 64% in laboratory conditions [29].

The qualitative and quantitative composition of protein concentrates of the studied algae species indicated they could be considered to be a protein source for both food products and feed additives. Only the digestibility and safety of these proteins can limit their use, and this is the goal of further research.

Nutritional supplements and feed additives based on microalgae are now widely used for complete feeds and prevention of many human and agricultural diseases. Their range is expanding annually, and the number of such additives is increasing. The advantages of microalgae amino acids over synthetic ones for humans and livestock are their healthiness, easy digestibility, and low toxicity. A limiting factor in introducing domestic microalgae amino acids into the production of dietary supplements and feed additives is the lack of information on the amino acid profile of microalgae and poor knowledge of their useful properties. First of all, this problem can be solved by studying the amino acid composition of microalgae and searching for promising methods for their extraction and purification for the production of dietary supplements and feed additives. The use of microalgae amino acids for the production of dietary supplements and feed additives for livestock is relevant from the point of view of improving the regional economy, improving the population’s quality of life, and improving the health of the nation in modern Russia.

## Figures and Tables

**Table 1:** The fractional composition of the protein complex obtained from the microalgae biomass.

Molecular Weight Range, kDa	The Relative Content of the Fraction, %				
	*Chlorella vulgaris*	*Arthrospira platensis*	*Nostoc* sp.	*Dunaliella salina*	*Pleurochrysis carterae*
227.0–225.0	9.0 ^a^	9.6 ^a^	8.4 ^b^	9.2 ^a^	9.8 ^a^
115.0–112.0	11.5 ^a^	11.4 ^a^	9.3 ^b^	10.8 ^a^	12.2 ^a^
70.0–67.0	12.0 ^a^	12.3 ^a^	14.5 ^b^	11.6 ^a^	11.8 ^a^
45.0–43.0	13.7 ^a^	13.9 ^a^	14.9 ^b^	14.3 ^b^	13.4 ^a^
35.0–33.0	8.2 ^a^	8.3 ^a^	10.3 ^b^	8.1 ^a^	8.0 ^a^
27.0–25.0	11.6 ^a^	10.8 ^a^	6.1 ^b^	11.6 ^a^	11.9 ^a^
21.0–20.0	11.5 ^a^	11.2 ^a^	12.1 ^a^	10.7 ^b^	12.0 ^a^
17.0–15.0	10.4 ^a^	10.8 ^a^	11.8 ^b^	12.4 ^b^	9.1 ^c^
15.0–13.0	12.1 ^a^	11.7 ^a^	12.6 ^a^	11.3 ^a^	11.8 ^a^

Values in a row followed by the same letter a, b or c do not differ significantly (*p* > 0.05) as assessed by the post hoc test (Duncan test). Average values are presented (*n* = 3).

**Table 2:** The fractional composition of the protein complex obtained from the microalgae biomass after the ultrafiltration process.

Molecular Weight Range, kDa	The Relative Content of the Fraction, %				
	*Chlorella vulgaris*	*Arthrospira platensis*	*Nostoc* sp.	*Dunaliella salina*	*Pleurochrysis carterae*
227.0–225.0	14.8 ^a^	15.4 ^a^	14.7 ^a^	16.2 ^b^	17.3 ^b^
115.0–112.0	17.6 ^a^	18.8 ^b^	16.5 ^a^	19.0 ^b^	21.5 ^c^
70.0–67.0	19.7 ^a^	21.1 ^a^	25.4 ^c^	23.4 ^c^	20.8 ^a^
45.0–43.0	9.8 ^a^	7.4 ^b^	9.8 ^a^	8.2 ^b^	7.6 ^b^
35.0–33.0	6.7 ^a^	6.2 ^a^	6.6 ^a^	6.8 ^a^	6.6 ^a^
27.0–25.0	7.6 ^a^	7.8 ^a^	3.7 ^b^	6.6 ^a^	6.9 ^a^
21.0–20.0	8.5 ^a^	7.7 ^b^	8.3 ^a^	6.4 ^c^	6.8 ^c^
17.0–15.0	7.6 ^a^	8.1 ^a^	7.4 ^a^	7.0 ^a^	5.8 ^b^
15.0–13.0	7.7 ^a^	7.5 ^a^	7.6 ^a^	6.4 ^b^	6.7 ^b^

Values in a row followed by the same letter a, b or c do not differ significantly (*p* > 0.05) as assessed by the post hoc test (Duncan test). Average values are presented (*n* = 3).

**Table 3:** The fractional composition of the protein complex obtained from the microalgae biomass after HPLC.

Molecular Weight Range, kDa	The Relative Content of the Fraction, %				
	*Chlorella vulgaris*	*Arthrospira platensis*	*Nostoc* sp.	*Dunaliella salina*	*Pleurochrysis carterae*
227.0–225.0	18.4 ^a^	19.1 ^b^	18.2 ^a^	19.2 ^b^	21.1 ^c^
115.0–112.0	27.6 ^a^	28.2 ^a^	26.4 ^a^	30.4 ^b^	29.5 ^b^
70.0–67.0	31.5 ^a^	31.6 ^a^	36.2 ^b^	32.1 ^a^	31.8 ^a^
45.0–43.0	4.8 ^a^	3.5 ^b^	4.4 ^a^	3.6 ^b^	3.3 ^b^
35.0–33.0	3.2 ^a^	2.7 ^a^	2.5 ^a^	2.9 ^a^	2.7 ^a^
27.0–25.0	3.1 ^a^	3.2 ^a^	1.5 ^b^	2.7 ^a^	2.9 ^a^
21.0–20.0	2.5 ^a^	2.6 ^a^	2.7 ^a^	1.8 ^a^	1.9 ^a^
17.0–15.0	4.6 ^a^	4.9 ^a^	3.8 ^a^	3.7 ^ab^	3.0 ^b^
15.0–13.0	4.3 ^a^	4.2 ^a^	4.3 ^a^	3.6 ^a^	3.8 ^a^

Values in a row followed by the same letter a, b or c do not differ significantly (*p* > 0.05) as assessed by post hoc test (Duncan test). Average values are presented (*n* = 3).

**Table 4:** Amino acid profile of proteins found in microalgae.

Amino Acid	Amino Acid Content, g/100 g Protein				
	*Chlorella vulgaris*	*Arthrospira platensis*	*Nostoc* sp.	*Dunaliella salina*	*Pleurochrysis carterae*
Alanine	10.82 ± 0.32 ^a^	11.48 ± 0.34 ^a^	9.88 ± 0.29 ^a^	10.99 ± 0.32 ^a^	11.51 ± 0.34 ^a^
Arginine	7.33 ± 0.21 ^a^	6.02 ± 0.18 ^a^	6.15 ± 0.18 ^a^	8.16 ± 0.24 ^a^	6.88 ± 0.20 ^a^
Aspartic acid	8.54 ± 0.25 ^a^	10.12 ± 0.30 ^b^	9.18 ± 0.27 ^a^	9.56 ± 0.28 ^ab^	9.19 ± 0.27 ^a^
Cysteine	1.47 ± 0.04 ^a^	1.94 ± 0.05 ^a^	1.54 ±0.04 ^a^	1.63 ± 0.04 ^a^	2.03 ± 0.06 ^a^
Glutamic acid	10.28 ± 0.30 ^a^	14.36 ± 0.43 ^b^	12.38 ± 0.37 ^a^	12.41 ± 0.37 ^a^	15.17 ± 0.45 ^b^
Glycine	7.14 ± 0.21 ^a^	5.25 ± 0.15 ^b^	6.54 ± 0.19 ^ab^	8.71 ± 0.26 ^a^	7.02 ± 0.21 ^a^
Histidine	1.52 ± 0.04 ^a^	2.19 ± 0.06 ^a^	2.01 ± 0.06 ^a^	1.73 ± 0.05 ^a^	1.89 ± 0.05 ^a^
Isoleucine	3.36 ± 0.10 ^a^	4.48 ± 0.13 ^a^	3.68 ± 0.11 ^a^	4.09 ± 0.12 ^a^	4.22 ± 0.12 ^a^
Leucine	8.41 ± 0.25 ^a^	9.81 ± 0.29 ^a^	9.41 ± 0.28 ^a^	9.58 ± 0.28 ^a^	9.93 ± 0.29 ^a^
Lysine	5.35 ± 0.16 ^a^	7.11 ± 0.21 ^b^	6.47 ± 0.19 ^a^	5.99 ± 0.17 ^a^	7.24 ± 0.21 ^b^
Methionine	2.52 ± 0.07 ^a^	1.93 ± 0.05 ^a^	2.23 ± 0.06 ^a^	2.79 ± 0.08 ^a^	2.41 ± 0.07 ^a^
Phenylalanine	6.17 ± 0.18 ^a^	7.85 ± 0.23 ^a^	7.15 ± 0.21 ^a^	6.98 ± 0.20 ^a^	7.69 ± 0.23 ^a^
Proline	5.08 ±0.15 ^a^	5.17 ± 0.15 ^a^	5.28 ± 0.15 ^a^	5.23 ± 0.15 ^a^	5.12 ± 0.15 ^a^
Serine	4.34 ± 0.13 ^a^	3.31 ± 0.09 ^a^	3.16 ± 0.09 ^a^	4.81 ± 0.14 ^a^	3.48 ±0.10 ^a^
Threonine	5.46 ± 0.16 ^a^	4.57 ± 0.13 ^b^	5.31 ± 0.15 ^a^	5.16 ± 0.15 ^a^	5.67 ± 0.17 ^a^
Tryptophan	0.21 ± 0.01 ^a^	1.16 ± 0.03 ^b^	1.02 ± 0.03 ^b^	0.18 ± 0.01 ^a^	1.14 ± 0.03 ^b^
Tyrosine	4.34 ± 0.13 ^a^	7.85 ± 0.23 ^b^	6.84 ± 0.20 ^b^	4.86 ± 0.14 ^a^	7.69 ± 0.23 ^b^
Valine	6.89 ± 0.20 ^a^	7.81 ± 0.23 ^a^	7.15 ± 0.21 ^a^	7.23 ± 0.21 ^a^	7.55 ± 0.22 ^a^

Values in a row followed by the same letter a or b do not differ significantly (*p* > 0.05) as assessed by the post hoc test (Duncan test). Average values are presented (*n* = 3).

## Data Availability

Not applicable.

## Sample Availability

Samples of the microalgae are available from the authors.

## Appendix A. Sequences of the 18S Ribosomal RNA Gene of the Studied Microorganism (Sample I)

TGTAGTCATATGCTTGTCTCAAAGATTAAGCCATGCATGTCTAAGTATAAACTGCTTTATACTGTGAAACTGCGAATGGCTCATTAAATCAGTTATAGTTTATTTGATGGTACCTACTACTCGGATACCCGTAGTAAATCTAGAGCTAATACGTGCGTAAATCCCGACTTCTGGAAGGGACGTATTTATTAGATAAAAGGCCGACCGGGCTCTGCCCGACTCGCGGTGAATCATGATAACTTCACGAATCGCATGGCCTTGTGCCGGCGATGTTTCATTCAAATTTCTGCCCTATCAACTTTCGATGGTAGGATAGAGGCCTACCATGGTGGTAACGGGTGACGGAGGATTAGGGTTCGATTCCGGAGAGGGAGCCTGAGAAACGGCTACCACATCCAAGGAAGGCAGCAGGCGCGCAAATTACCCAATCCTGACACAGGGAGGTAGTGACAATAAATAACAATACTGGGCCTTTTCAGGTCTGGTAATTGGAATGAGTACAATCTAAACCCCTTAACGAGGATCAATTGGAGGGCAAGTCTGGTGCCAGCAGCCGCGGTAATTCCAGCTCCAATAGCGTATATTTAAGTTGCTGCAGTTAAAAAGCTCGTAGTTGGATTTCGGGTGGGGCCTGCCGGTCCGCCGTTTCGGTGTGCACTGGCAGGGCCCACCTTGTTGCCGGGGACGGGCTCCTGGGCTTCACTGTCCGGGACTCGGAGTCGGCGCTGTTACTTTGAGTAAATTAGAGTGTTCAAAGCAGGCCTACGCTCTGAATACATTAGCATGGAATAACACGATAGGACTCTGGCCTATCCTGTTGGTCTGTAGGACCGGAGTAATGATTAAGAGGGACAGTCGGGGGCATTCGTATTTCATTGTCAGAGGTGAAATTCTTGGATTTATGAAAGACGAACTACTGCGAAAGCATTTGCCAAGGATGTTTTCATTAATCAAGAACGAAAGTTGGGGGCTCGAAGACGATTAGATACCGTCCTAGTCTCAACCATAAACGATGCCGACTAGGGATCGGCGGATGTTTCTTCGATGACTCCGCCGGCACCTTATGAGAAATCAAAGTTTTTGGGTTCCGGGGGGAGTATGGTCGCAAGGCTGAAACTTAAAGGAATTGACGGAAGGGCACCACCAGGCGTGGAGCCTGCGGCTTAATTTGACTCAACACGGGAAAACTTACCAGGTCCAGACATAGTGAGGATTGACAGATTGAGAGCTCTTTCTTGATTCTATGGGTGGTGGTGCATGGCCGTTCTTAGTTGGTGGGTTGCCTTGTCAGGTTGATTCCGGTAACGAACGAGACCTCAGCCTGCTAAATAGTCACGGTTGGCTCGCCAGCCGGCGGACTTCTTAGAGGGACTATTGGCGACTAGCCAATGAAGCATGAGGCAATAACAGGTCTGTGATGCCCTTAGATGTTCTGGGCCGCACGCGCGCTACACTGATGCATTCAACGAGCTTAGCCTTGGCCGAGAGGCCCGGGTAATCTTTGAAACTGCATCGTGATGGGGATAGATTATTGCAATTATTAATCTTCAACGAGGAATGCCTAGTAAGCGCAAGTCATCAGCTTGCGTTGATTACGTCCCTGCCCTTTGTACACACCGCCCGTCGCTCCTACCGATTGGGTGTGCTGGTGAAGTGTTCGGATTGGCGACCGGGGGCGGTCTCCGCTCTCGGCCGCCGAGAAGTTCATTAAACCCTCCCACCTAGAGGAAGGAGAAGTCGTAACAAGGTTTCCGTAGGTGAACCTGCGGAAGGATCA

## Appendix B. Sequences of the 18S Ribosomal RNA Gene of the Studied Microorganism (Sample II)

AGAGTTTGATCCTGGCTCAGGATGAACGCTGGCGGTCTGCTTAACACATGCAAGTCGAACGGGCTCTTCGGAGCTAGTGGCGGACGGGTGAGTAACACGTGAGAATCTGGCTCCCGGTCGGGGACAACAGAGGGAAACTTCTGCTAATCCCGGATGAGCCGAAAGGTAAAAGATTTATCGCCGGGAGATGAGCTCGCGTCTGATTAGCTAGTTGGTGAGGTAAAGGCTCACCAAGGCGACGATCAGTAGCTGGTCTGAGAGGATGATCAGCCACACTGGGACTGAGACACGGCCCAGACTCCTACGGGAGGCAGCAGTGGAGAATTTTCCGCAATGGGCGCAAGCCTGACGGAGCAAGACCGCGTGGGGGAGGAAGGCTCTTGGGTTGTAAACCCCTTTTCTCAAGGAAGAACACAATGACGGTACTTGAGGAATAAGCCTCGGCTAACTCCGTGCCAGCAGCCGCGGTAATACGGAGGAGGCAAGCGTTATCCGGAATGATTGGGCGTAAAGCGTCCGTAGGTGGCAGTTCAAGTCTGCTGTCAAAGACAGTAGCTCAACTACTGAAAGGCAGTGGAAACTGAACAGCTAGAGTACGGTAGGGGCAGAGGGAATTCCCGGTGTAGCGGTGAAATGCGTAGATATCGGGAAGAACACCGGTGGCGAAAGCGCTCTGCTGGGCCGTAACTGACACTGAGGGACGAAAGCTAGGGGAGCGAATGGGATTAGATACCCCAGTAGTCCTAGCCGTAAACGATGGAAACTAGGTGTAGCCTGTATCGACCCGGGCTGTGCCGAAGCTAACGCGTTAAGTTTCCCGCCTGGGGAGTACGCACGCAAGTGTGAAACTCAAAGGAATTGACGGGGGCCCGCACAAGCGGTGGAGTATGTGGTTTAATTCGATGCAACGCGAAGAACCTTACCAGGGCTTGACATGTCCGGAATCTTGGTGAAAGCCGAGAGTGCCTTCGGGAGCCGGAACACAGGTGGTGCATGGCTGTCGTCAGCTCGTGTCGTGAGGTGTTGGGTTAAGTCCCGCAACGAGCGCAACCCTCGTCCTTAGTTGCCATCATTCAGTTGGGCACTTTAGGGAGACTGCCGGTGACAAACCGGAGGAAGGTGGGGATGACGTCAAGTCATCATGCCCCTTACGTCCTGGGCTACACACGTACTACAATGGGGGGGACAAAGGGTAGCCAAGACGCGAGTCTGAGCCAATCCCGTAAACCTCTCCTCAGTTCAGATTGCAGGCTGCAACTCGCCTGCATGAAGGAGGAATCGCTAGTAATCGCAGGTCAGCATACTGCGGTGAATCCGTTCCCGGGCCTTGTACACACCGCCCGTCACACCATGGAAGTTAGCCACGCCCGAAGTCGTTACTCTAACCGTTCGCGGAGGAGGATGCCGAAGGCAGGGCTGATGACTGGGGTGAAGTCGTAACAAGGTAGCCGTACCGGAAGGTGTGGCTGGATCACCTCCTTTTTAGGGAGACCTACTTCGAGATATCGCGCCTTAACAACTATAGCCGTGTCTTGAGGTCATCCTTAGGTCGGATGGGGCGGTCAGAGAGCTTTCAAACTTTAGGGTTCGTGTTATGGGCTATTAGCTCAGGTGGTTAGAGCGCACCCCTGATAAGGGTGAGGTCCCTGGTTCAAGTCCAGGATGGCCCACATCCACCCCAAACTGGGGGTATAGCTCAGTTGGTAGAGCGCTGCCTTTGCACGGCAGAAGTCAGCGGTTCGAGTCCGCTTACCTCCACTCTCCTTTGTGATGGTGCTAGTTGGGGTGAGATGAGATGAGATGACCTCTGATAGATAATTTATCACTGTACAGCTCCTAAATCTTTAGATGTTAGTCTGAGATTGGATAGCTGGACATCTGTTCCAGTCAGAACCTTGAAAACTGCATAGAGAAAAGCATAATGGTGTAGGAAAACGTCGTAAAGACAATTCCAATGTAGGTCAAGCTACAAAGGGCTAACGGTGGAACCTAGGCACACAGAGCGGCCGCAAA

## Appendix C. Sequences of the 18S Ribosomal RNA Gene of the Studied Microorganism (Sample III)

TGAGTTTGATCCTGGCTCAGGATGAACGCTGGCGGTATGCTTAACACATGCAAGTCGAACGGTGTCTTTCGGACACAGTGGCGGACGGGTGAGTAACGCGTGAGAATCTGGCTCTAGGTCTGGGACAACCACTGGAAACGGTGGCTAATACCGGATGTGCCCTTCGGGGTGAAAGGTTAACTGCCTGGAGATGAGCTCGCGTCTGATTAGCTAGTTGGGAAGTGTTCAAGTGGACTCCCAAGGCGACGATCAGTAGCTGGTCTGAGAGGACGATCAGCCACACTGGGACTGAGACACGGCCCAGACTCCTACGGGAGGCAGCAGTGGGGAATTTTCCGCAATGGGCGAAAGCCTGACGGGAGCAATACCGCGTGAGGGAGGAAGGCTCTTGGGTCGTAAACGCTCTTTTCTCAGGGAAGAACACAATGACGGTACCTGAGGAATAAGCATCGGCTAACTCCGTGCCAGCAGCCGCGGTAATACGGAGGATGCAAGCGTTATCCGGAATGATTGGGCGTAAAGCGTCCGCAGGTGGCAATGTAAGTCTGCTGTTAAAGAGTCTAGCTCAACTAGATAAAAGCAGTGGAAACTACATAGCTAGAGTGCGTTCGGGGCAGAGGGAATTCCTGGTGTAGCGGTGAAATGCGTAGAGATCAGGAAGAACACCAGTGGCGAAGGCGCTCTGCTAGGCCGTAACTGACACTGAGGGACGAAAGCTAGGGGAGCGAATGGGATTAGATACCCCAGTAGTCCTAGCCGTAAACGATGGATACTAGGCGTGGCTTGTATCGACCCGAGCCGTGCCGTAGCTAACGCGTTAAGTATCCCGCCTGGGGAGTACGCCGGCAACGGTGAAACTCAAAGGAATTGACGGGGGCCCGCACAAGCGGTGGAGTATGTGGTTTAATTCGATGCAACGCGAAGAACCTTACCAAGGCTTGACATGTCGCGAATCTTCTCGAAAGGGAAGAGTGCCTTCGGGAGCGCGAACACAGGTGGTGCATGGCTGTCGTCAGCTCGTGTCGTGAGATGTTGGGTAAGTCCCGCAACGAGCGCAACCCTCGTTTTAGTTGCCAGCATTAAGTTGGGCACTCTAGAGAGACTGCCGGTGACAAACCGGAGGAAGGTGGGGATGACGTCAAGTCAGCATGCCCCTTACGCCTTGGGCTACACACGTACTACAATGCTCCGGACAGAGGGCAGCAAGCATGCGAATGCAAGCAAATCCCGTAAACCGGAGCTCAGTTCAGATCGCAGGCTGCAACTCGCCTGCGTGAAGGAGGAATCGCTAGTAATTGCAGGTCAGCATACTGCAGTGAATTCGTTCCCGGGCCTTGTACACACCGCCCGTCACACCATGGAAGCTGGTAGTGCCCGAAGTCATTACTCCAACTTTTCGGAGAGGAGGATGCCTAAGGCAGGACTGGTGACTGGGGTGAAGTCGTAACAAGGTAGCCGTACCGGAAGGTGTGCTGGGGATCACTC

## Appendix D. Sequences of the 18S Ribosomal RNA Gene of the Studied Microorganism (Sample IV)

CTGGTTGATCCTGCCAGTAGTCATATGCTTGTCTCAAAGATTAAGCCATGCATGTCTAAGTATAAACTGCTTATACTGTGAAACTGCGAATGGCTCATTAAATCAGTTATAGTTTATTTGATGGTACCTTTACTCGGATAACCGTAGTAATTCTAGAGCTAATACGTGCGTAAATCCAGACTTCTGGAAGGGACGTATTTATTAGATAAAAGGCCAGCCGGGCTTGCCCGACTCTTGGCGAATCATGATAACTTCACGAATCGCACGGCTTTATGCCGGCGATGTTTCATTCAAATTTCTGCCCTATCAACTTTCGATGGTAGGATAGAGGCCTACCATGGTGGTAACGGGTGACGGAGGATTAGGGTTCGATTCCGGAGAGGGAGCCTGAGAAACGGCTACCACATCCAAGGAAGGCAGCAGGCGCGCAAATTACCCAATCCCAACACGGGGAGGTAGTGACAATAAATAACAATACCGGGCATTTTTGTCTGGTAATTGGAATGAGTACAATCTAAATCCCTTAACGAGTATCCATTGGAGGGCAAGTCTGGTGCCAGCAGCCGCGGTAATTCCAGCTCCAATAGCGTATATTTAAGTTGTTGCAGTTAAAAAGCTCGTAGTTGGATTTCGGGTGGGTTGTAGCGGTCAGCCTTTGGTTAGTACTGCTACGGCCTACCTTTCTGCCGGGGACGAGCTCCTGGGCTTAACTGTCCGGGACTCGGAATCGGCGAGGTTACTTTGAGTAAATTAGAGTGTTCAAAGCAAGCCTACGCTCTGAATACATTAGCATGGAATAACACGATAGGACTCTGGCTTATCTTGTTGGTCTGTAAGACCGGAGTAATGATTAAGAGGGACAGTCGGGGGCATTCGTATTTCATTGTCAGAGGTGAAATTCTTGGATTTATGAAAGACGAACTTCTGCGAAAGCATTTGCCAAGGATGTTTTCATTAATCAAGAACGAAAGTTGGGGGCTCGAAGACGATTAGATACCGTCGTAGTCTCAACCATAAACGATGCCGACTAGGGATTGCCAGGTGTTTCGTTGATGACCCTGCCAGCACCTTATGAGAAATCAAAGTTTTTGGGTTCCGGGGGGAGTATGGTCGCAAGGCTGAAACTTAAAGGAATTGACGGAAGGGCACCACCAGGCGTTAACTTAGCAGCAAGCTCAGCGCCTCAAAGTCGAAGGGAAACCTTTGGCTAGTATCTGGGTGTAGATTTCACCTAAGTGCAACACTGTTCAAATTGCGGGAAAGCCCTAAAGCTTTGCTAACCAAGCTGTCCTAGAAATGGGATGGTGGCCAGGTGAAAGACCTTGGGTACGGTAAAATCAGCAAAGATGCAACAATGGGCAATCCGCAGCCAAGCTCCTACGGGCTGTCAAAGCCTATGGAGAAGGTTCAGAGACTAAATGGCAGTGGGCAAGCATGGCAATGCTTGCTTAAGATATAGTCCGTCCCAGCTGAGAAGCTGCCTATGAGAGGAATGCCGTAAGGCAGGAGAGCTAATAGGAAGTAAGTGTCTTTAATCAACTTACTTGGATTCCACGGGAGCCTGCGGCTTAATTTGACTCAACACGGGAAAACTTACCAGGTCCAGACACGGGGAGGATTGACAGATTGAGAGCTCTTTCTTGATTCTGTGGGTGGTGGTGCATGGCCGTTCTTAGTTGGTGGGTTGCCTTGTCAGGTTGATTCCGGTAACGAACGAGACCTCAGCCTGCTAAATAGTCACGTCTACCTCGGTAGGCGCCTGACTTCTTAGAGGGACTATTGGCGTTTAGCCAATGGAAGTGTGAGGCAATAACAGGTCTGTGATGCCCTTAGATGTTCTGGGCCGCACGCGCGCTACACTGATGCATTCAACGAGCCTATCCTTGGCCGAGAGGTCCGGGTAATCTTTGAAACTGCATCGTGATGGGGATAGATTATTGCAATTATTAGTCTTCAACGAGGAATGCCTAGTAAGCGCGAGTCATCAGCTCGCGTTGATTACGTCCCTGCCCTTTGTACACACCGCCCGTCGCTCCTACCGATTGGGTGTGCTGGTGAAGTGTTTGGATCGGTACCAATGGGGGGAAACCTCTGTTGGTACTGAGAAGAACATTAAACCCTCCCACCTAGAGGAAGGAGAAGTCGTAACAAGGTTTCCGTAGGTGAACCT GCAGAAGGATCA

## Appendix E. Sequences of the 18S Ribosomal RNA Gene of the Studied Microorganism (Sample V)

AACCTGGTTGATCCTGCCAGTAGTCATATGCTTGTCTCAAAGATTAAGCCATGCATGTCTAAGTATAAGCGAGTATACAGTGAAACTGCGAATGGCTCATTAAATCAGTTATGGTTTATTTGATGGTACCTTACTACTTGGATACCCGTAGTAATTCTAGAGCTAATACATGCAGGAGTTCGCTGGTTCKTGCGCCGCGATGTATTTATTAGATAAGAGACCAACCCGCCTTGTGCGGTTGCGTGCCGAGTCATAATAACTGTTCGAATCGCATGGCTCTGACGCCGGCGATGGTTCATTCAAGTTTCTGCCCTATCAGCTTTCGATGGTAGGATAGAGGCCTACCATGGCGTTAACGGGTAACGGAGAATTAGGGTTCGATTCCGGAGAGGGAGCCTGAGAGATGGCTACCACATCCAAGGAAGGCAGCAGGCGCGTAAATTGCCCGAATCCTGACACAGGGAGGTAGTGACAAGAAATAACAATACAGGGCCATCTTGGTCTTGTACTTGGAATGAGTACAATTTACATCTCTTCACGAGGATCAATTGGAGGGCAAGTCTGGTGCCAGCAGCCGCGGTAATTCCAGCTCCAATAGCGTATATTAAAGTTGTTGCAGTTAAAACGCTCGTAGTCGGATTTCGGGTCGGTTGCGCCGGTCTGCCGATGGGTATGCACTGGCGGAGTCGTCCTTTCTTCCGGAGACCGGGCCTCCTCTTAGCTGAGCGGGTTCGGGAGACGGATCGTTTACTTTGAAAAAATCAGAGTGTTTCAAGCAGGCAGCTCGCTCTTGCATGGATTAGCATGGGATAATGAAATAGGACTCTGGTGCTATTTTGTTGGTTTCGAACACCGGAGTAATGGTCAACAGGGACAGTCAGGGGCACTCGTATTCCGCCGAGAGAGGTGAAATTCTCAGACCAGCGGAAGACGAACCACTGCGAAAGCATTTGCCAGGGATGTTTTCACTGATCAAGAACGAAAGTTAGGGGATCGAAGACGATCAGATACCGTCGTAGTCTTAACCATAAACCATGCCGACTAGGGATTGGAGGCTGTTCCTTTTGTGACTCCTTCAGCACCTTTCGGGAAACTAAAGTCTTTGGGTTCCGGGGGGAGTATGGTCGCAAGGCTGAAACTTAAAGGAATTGACGGAAGGGCACCACCAGGAGTGGAGCCTGCGGCTTAATTTGACTCAACACGGGGAAACTTACCAGGTCCAGACATTGTGAGGATTGACAGATTGAGAGCTCTTTCTTGATTCGATGGGTGGTGGTGCATGGCCGTTCTTAGTTGGTGGAGTGATTTGTCTGGTTAATTCCGTTAACGAACGAGACCGCAGCCTGCTAAATAGTTTCGCGAACACTCCGTTGGCGTTGAGCTTCTTAGAGGGACAACTTGTCTTCAACAAGTGGAAGTTTGCGGCAATAACAGGTCTGTGATGCCCTTAGATGTTCTGGGCCGCACGCGCGCTACACTGATGCATTCAGCGAGTCTCTTCCCTTGGCCGAGAGGTCCGGGTAATCTTGTGAACTTGCATCGTGATGGGGATAGATTATTGCAATTATTAATCTTCAACGAGGAATTCCTAGTAAGCGCATGTCATCAGCGTGCGTTGATTACGTCCCTGCCCTTTGTACACACCGCCCGTCGCTCCTACCGATTGAATGATCCGGTGAGGCCCCCGGACTGTGGCAACGTGGCTGGTGTTCCAGCCGCGATGCCGCGGGAAGTTGTCCAAACCTTATCATTTAGAGGAAGGAGAAGTCGTAACAAGGTTTCCGTAGGTGAACCTGCAGAAGGATCAA
